# An Intercategorical Ecology of Lead Exposure: Complex Environmental Health Vulnerabilities in the Flint Water Crisis

**DOI:** 10.3390/ijerph18052217

**Published:** 2021-02-24

**Authors:** Raoul S. Liévanos, Clare R. Evans, Ryan Light

**Affiliations:** Department of Sociology, University of Oregon, Eugene, OR 97403-1291, USA; cevans@uoregon.edu (C.R.E.); light@uoregon.edu (R.L.)

**Keywords:** lead, drinking water, Flint, Michigan, ecology, spatial analysis, intersectionality, race, gender, family, environmental justice

## Abstract

In 2014, city and state officials channeled toxic water into Flint, Michigan and its unevenly distributed and corroding lead service lines (LSLs). The resulting Flint water crisis is a tragic example of environmental racism against a majority Black city and enduring racial and spatial disparities in environmental lead exposures in the United States. Important questions remain about how race intersected with other established environmental health vulnerabilities of gender and single-parent family structure to create unequal toxic exposures within Flint. We address this question with (1) an “intercategorical ecology” framework that extends the “racial ecology” lens into the complex spatial and demographic dimensions of environmental health vulnerabilities and (2) a multivariate analysis using block-level data from the 2010 U.S. decennial census and a key dataset estimating the LSL connections for 56,038 land parcels in Flint. We found that blocks exposed to LSLs had, on average, higher concentrations of single-parent white, Black, and Latinx families. However, logistic regression results indicate that the likelihood of block exposure to LSLs was most consistently and positively associated with the percentage of single-father Black and single-mother Latina families, net of other racialized and gendered single-parent family structures, socioeconomic status, and the spatial concentration of LSL exposure.

## 1. Introduction

### 1.1. Background

Lead is a neurotoxin capable of adversely affecting human health throughout the life course. Exposure is particularly problematic if it occurs during fetal and childhood development due to its effects on a variety of developing body systems, including the nervous, hematopoietic, and renal systems [1,2,3]. Lead pipes are among the primary contemporary sources of lead exposure in the United States [2,3,4]. They have been an important component of U.S. cities’ drinking water distribution systems for at least 150 years despite wide recognition of the dangers they pose to human health [5]. Exposures to harmful lead levels are disproportionately experienced among inner-city Black and/or Latinx children who live in substandard housing in the United States [4]. However, case studies of Detroit, Michigan [6], and Chicago, Illinois [7], demonstrate that the “racial ecology” (i.e., the racial and spatial patterning) of lead exposure manifests differently across cities.

The Flint water crisis (FWC) is a well-known tragedy regarding elevated toxic exposures to lead in the water supply in Flint, Michigan, that began in April 2014. It resulted from a “cost-saving” switch in the water supply from Lake Huron to the Flint River by city leaders and state emergency managers. Water drawn from the Flint River was more corrosive, incorrectly treated, and contributed to increased water lead levels (WLLs) and blood lead levels (BLLs) for many residents connected to Flint’s lead service lines (LSLs), that is, to the pipes that bring the city’s water from the main into people’s homes [8,9]. Researchers attribute the FWC to the intertwined histories of systemic racism and segregation, disinvestment, and political isolation and fragmentation in both the Flint metropolitan area and the state of Michigan that placed much of Flint’s predominantly Black population (56.6 percent in 2010) at heightened risk of exposure to the contaminated drinking water supply [10,11]. The case of Flint is generally accepted as a clear manifestation of environmental racism with adverse environmental health consequences for all of Flint’s diverse and marginalized residents and households linked to the city’s aging drinking water system [12].

Yet, not all residents of Flint were affected by the crisis to the same degree. Research paints a varied picture of the racial ecology of lead exposure within Flint during the crisis. Using regression techniques, Kennedy et al. [13] found racial disparities in elevated BLLs in young children, but those disparities were mediated by other factors, like age and season. Using geographic information systems (GIS), Hanna–Attisha et al. [8] found robust racial and spatial disparities in exposure at the census block group level, while Sadler, LaChance, and Hanna–Attisha [14] found that racially integrated block groups were more vulnerable than isolated Black block groups. However, a consistent finding across these prior studies [8,14] is that the spatial concentration of single-parent families and indicators of socioeconomic disadvantage (i.e., poverty and low educational attainment) were positively correlated with elevated lead exposure levels during the FWC.

In this article, we address one key lingering question about the FWC that has broader implications for understanding the spatial and demographic patterning of lead exposure in U.S. cities, as well as environmental health vulnerabilities more generally. We ask the following question: controlling for socioeconomic status and the spatial concentration of LSL exposure, how did race intersect with other established environmental health vulnerabilities, specifically, gender and single-parent family structure, to create unequal toxic exposures within Flint?

We address this question with high-resolution census block-level data from two sources. We use a detailed assessment by the University of Michigan–Flint (UMF) GIS Center [15] of Flint’s LSLs—a primary mechanism of lead exposure when coupled with corrosive water during the FWC. We merge these data with population and housing data from the 2010 U.S. decennial census. Our merged dataset allows us to make explicit, focused, and innovative assessments of claims made in previous research regarding the spatial and demographic dimensions of the FWC [8,10,11,14]. Our analysis controls for available census block-level indicators of socioeconomic status and the spatial concentration of LSL exposure in neighboring census blocks which capture dimensions of environmental health vulnerability found in prior racial ecology of lead exposure and environmental inequality research.

Using the case of Flint, we advance an “intercategorical ecology” framework that synthesizes the racial ecology lens [4,6,7] with intercategorical approaches to identifying intersecting social [16] and environmental inequalities [17,18,19,20]. We find that blocks exposed to LSLs had, on average, higher concentrations of single-parent white, Black, and Latinx families. However, logistic regression results indicate that the likelihood of block exposure to LSLs was most consistently and positively associated with the percentage of single-father Black and single-mother Latina families, net of other racialized and gendered single-parent family structures, socioeconomic status, and the spatial concentration of LSL exposure. We conclude the article with a discussion of its scholarly and practical implications.

### 1.2. Toward an Intercategorical Ecology of Lead Exposure

Our analysis of the demographic and spatial dynamics of lead exposure during the FWC integrates key insights from the intersectionality literature. Intersectionality, as defined by Hill Collins [21] (p. 2), “references the critical insight that race, class, gender, sexuality, ethnicity, nation, ability, and age operate not as unitary, mutually exclusive entities, but rather as reciprocally constructing phenomena.” It has roots in U.S. Black feminism of the 1960s and 1970s, race/class/gender analyses within women’s studies in the 1980s, and critical legal scholarship [22,23] on the systemic disadvantages experienced by women of color [21,24]. In her now classic statement on the methodological implications of intersectionality theory, McCall [16] distinguished early “intracategorical” approaches focused on within-group differences from “intercategorical” approaches that attempt to analyze the relationships between groups. Earlier studies of intersecting environmental health vulnerabilities feature an intracategorical approach by focusing on “particular social groups at neglected points of intersection” [16] (p. 1774). For example, case studies of El Paso County, Texas [25], and of Miami, Florida [26], document unequal cancer risks associated with ambient air toxic exposures within various Latinx communities. Likewise, Grineski et al. [27] examined nationwide disparities in carcinogenic air pollution exposure for various Asian American groups.

In contrast, we build on a growing body of research [17,18,19,20] that uses an intercategorical approach to examine intersecting environmental health vulnerabilities. For example, Liévanos [17] finds that the intersection of race, class, immigrant status, linguistic ability, and female-headed households affects the spatial distribution of air-toxic lifetime cancer risk. In building on such work, we draw attention to the “relationships of inequality among social groups and changing configurations of inequality along multiple and conflicting dimensions” [16] (p. 1773). We propose an intercategorical ecology that recognizes how the differential geographic distribution of intersecting groups and identities—based on historical and contemporary systems of power—impacts environmental inequalities. Our approach thus moves beyond the central focus on the distribution of racial groups and/or on the distribution of subpopulations within a single racial group as a predictor of environmental inequality that characterizes prior research.

Specifically, we focus our intercategorical inquiry on how race, gender, and single-parent family structure intersect and shape the spatial and demographic patterning of lead exposure during the FWC. Previous research on the FWC has only considered some of these axes of social division and environmental health vulnerability independently, providing mixed results on the extent of racial disparities in exposure while noting heightened vulnerabilities associated with the spatial concentration of single-parent families [8,14]. Likewise, the broader U.S.-based environmental inequality literature finds significant inequalities in air pollution exposures associated with race [28], as well as with gendered family structures, whereby single-mother families experience disproportionate exposures when compared to single-father families [29,30]. Those findings corroborate other sociological research on the important role that gendered family structure plays in the reproduction of racial and gender inequalities, especially for single-mother families in the United States [31].

Gendered racial formation theory offers insights into why the spatial concentration of differentially gendered and racialized family formations, such as the extent of racially marginalized single-mother family households in a neighborhood, may be associated with heightened risk of exposure to environmental health hazards. Gendered racial formation theory illuminates how dominant social actors deploy “controlling images” or stigmatizing and oppressive social constructions that help to produce and legitimate complex inequalities that are experienced differentially given a group’s subordinated gender and racial status [24,32,33,34,35]. White, male elites have historically managed controlling images and benefited from such systems of oppression within the United States [34]. Particularly strong controlling images have historically targeted single Black mothers—and more recently focused on single Latina mothers—as gendered and racialized deviants who are not deemed deserving of a broad array of social service provisions and environmental protections in the United States [24,35,36]. Or, at a minimum, these groups’ and their families’ heightened vulnerability to environmental hazard is easily ignored.

Controlling images can also be “spatial strategies” that inscribe intercategorical hierarchies into the urban landscape [36,37,38,39,40]. During the 1950s, federal loan underwriting procedures in the United States prioritized white, male, and married borrowers for low-interest mortgages, resulting in their exclusive access to new suburbs with “single-family homes” and “family-friendly” amenities (i.e., schools, churches, parks, and playgrounds) [40,41]. These policies, combined with local segregationist ideologies and real estate practices, then excluded unmarried people, especially women, Black and Latinx people, and their children from suburban space and channeled them into low-income, deteriorating, crowded, and devalued settlement spaces of inner cities [40,42,43,44,45,46,47,48]. In other words, race, gender, and family structure (e.g., single-parent families) intersect within United States housing markets and reflect historical systems of power. Further, those intersecting axes of social division act as resources and risks, such as exposure to environmental health hazards, which are often unevenly distributed in the United States. U.S. metropolitan areas are thus marked by the inscription of multiply marginalized social hierarchies in physical space with implications for people given their contemporary social and spatial location within the “matrix of domination” [24,36]. To gain a more complete understanding of inequality and multiple marginalization, we must develop intercategorical ecological models that account for how the “changing configurations of inequality” are embedded in geographic space [16] (p. 1773). An intercategorical ecological model is necessary to understand the spatial and demographic complexities of the FWC.

### 1.3. The Case of Flint

Flint is an ideal case to develop and evaluate an intercategorical ecology approach to environmental health hazard exposure that focuses on the relationship between race, gender, and single-parent family structure. Single-parent families were the majority (60.92 percent) of the 23,949 family households enumerated in Flint in 2010. Previous research describes the positive association between the spatial concentration of single-parent families and rates of lead exposure (and poisoning) during the FWC [8,14]. Single-mother families were the predominant family structure in Flint by 2010, as they represented 48.95 percent of all families and 80.35 percent of all single-parent families. In particular, Black single-mother families were the predominant racialized and gendered family structure, representing 34.32 percent of all families, 56.34 percent of single-parent families, and 70.12 percent of all single-mother families.

Flint’s racialized and gendered family structure overlays onto a history of concentrated poverty and multiple marginalization among women, especially women of color. The resulting “feminization of poverty” within Flint during its economic crises of the 1970s and 1980s continued into 2010 with women “accounting for 54 percent of the city’s residents living below the federal poverty level” [47] (p. 273). Further, labor market discrimination from automobile producers and other major local employers relegated many of Flint’s Black women to low-paying jobs and limited opportunities for independent wealth accumulation [47]. These dynamics reflect the national pattern of single-parent and single-mother family prevalence amongst the systemically marginalized and segregated Black population [31,49].

In addition, Flint had 3.9 percent Latinx composition in 2010 (the majority of which, 3.0 percent, was of Mexican origin). During “The Fall of Flint” between 1970 and 2010 [47], Flint had above-average increases in the neighborhood concentration of Latinx residents (from 1.91 to 10.40 percent). Some of the highest increases in Latinx composition occurred in Flint’s impoverished east and southwest neighborhoods, which also experienced increases in single-parent families since 1970 (see Appendix A) and elevated WLLs and BLLs during the FWC [8].

Multiple marginalization at the intersection of race, gender, and single-parent family structure may have contributed to particular environmental health vulnerabilities for Black single-father families during the FWC. Urban [50,51], intersectionality [24,33,34], and environmental justice scholars [52] all support Young’s [53] (p. 28) contention that Black men are pejoratively depicted as “invested in delinquency and indecency”, “hopeless”, and, therefore, segregated from channels of sociospatial mobility and environmental protection while being socially controlled with increased police scrutiny within U.S. metropolitan areas. Furthermore, public health research indicates that the significant political, economic, racialized, and gendered stressors experienced by Black men—particularly fathers committed to supporting their family under such conditions—can contribute to their multiple marginalization in a community and adversely affect their overall health and wellbeing [54]. Focus group-based community health research in Flint indicates that Black men and fathers in the city face elevated risks of mortality, heart disease, stroke, cancer, and other health ailments when compared to Black women and white men and women throughout the Flint metropolitan area [55]. In our analysis, one of the inequalities we evaluate in particular is the extent to which the spatial concentration of single-father Black families is associated with elevated likelihood of exposure to LSLs. Such families were less prevalent in Flint as of 2010, representing only 6.58 percent of all families and 10.80 percent of all single-parent families. However, they were the majority (54.97 percent) of all single-father families in 2010 within Flint.

### 1.4. Research Aims

This study has two main aims. First, we examine the association between increasing concentrations of white, Black, and Latinx single-parent families in census blocks and the likelihood of exposure to LSL-connected parcels. Based on the existing literature, we hypothesize that a higher percent of nonwhite single-parent families will be positively associated with increased likelihood of block exposure to LSL-connected parcels. Our analysis is inclusive of Latinx families because we argue it is imperative to move beyond the Black–white binary characteristic of previous accounts of Flint and its water crisis [8,14,47] to a “relational” [56] and multigroup notion of intersecting environmental health vulnerabilities [17,18,19,20] that are inscribed in Flint’s diverse residential settlements.

While the first aim therefore involves analysis of the environmental vulnerability of racialized single-parent family structures, our second research aim builds on this to include consideration of how racialized single-parent families are also gendered. We examine the intersection of gendered householders with race and family structure to consider whether particular intersections are at especially high risk of exposure to LSL. Based on the prior literature and the Flint case context that we reviewed above, we hypothesize that higher concentrations of single-mother Black and Latina families, as well as single-father Black families, in census blocks will be associated with increased likelihood of exposure to LSL-connected parcels.

## 2. Materials and Methods

### 2.1. Unit of Analysis

We use census blocks as our primary unit of analysis [57]. Census blocks “are statistical areas bounded by visible features, such as streets, roads, streams, and railroad tracks, and by nonvisible boundaries, such as selected property lines and city, township, school district, and county limits and short line-of-sight extensions on all sides by streets” [58] (p. A-10). Larger levels of aggregation like census block groups [7,8,14] or tracts [6] have been used in prior ecological studies of lead exposure. In contrast, we use census blocks because they are the smallest geographic units available from the U.S. census to examine aggregate-level residential settlement patterns [59] and exposure to environmental health hazards [60] and, therefore, provide the most refined available unit of analysis connecting census data to urban space.

### 2.2. Dependent Variable

**Figure 1:**
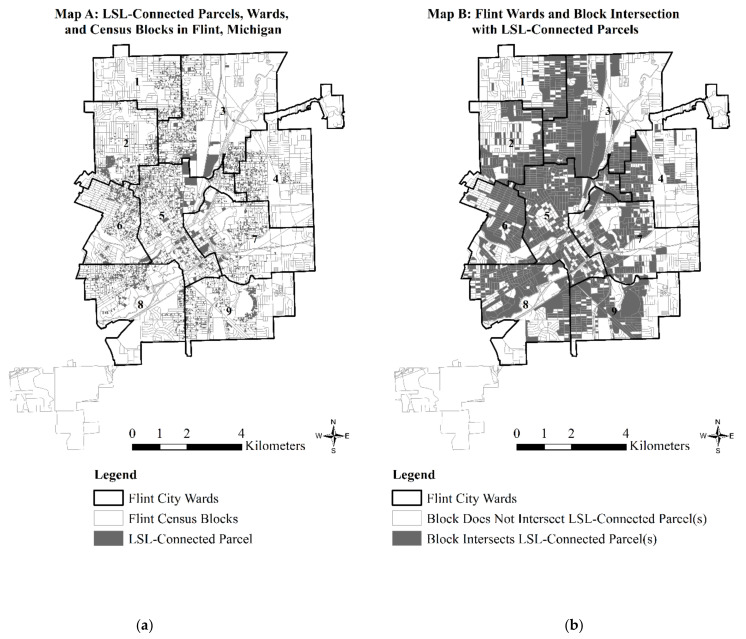
(**a**) Parcels with lead service line (LSL) connections as of 7 November 2016, ward boundaries, and census blocks in Flint, Michigan; and (**b**) wards and block intersection with LSL-connected parcels.

Our dependent variable comes from data collected by the UMF GIS Center [15]. In contrast to the smaller-scale regulatory surveys of LSLs in Flint [61], the UMF GIS Center [15] estimated the type of water service line connection for 56,038 land parcels in Flint, current as of 7 November 2016. Land parcels tend to be small—representing geographic space that hosts a single dwelling or apartment building. Figure 1, Map A, displays the parcels with LSL connections overlaid on census blocks within the 2010 Flint City boundaries [57] and digitized ward boundaries from city [62] and county [63] sources. We present ward boundaries below and throughout to maintain greater comparability with prior research [8].

Our dependent variable is a binary measure of *block intersection with LSL-connected parcels* (1 = yes, 0 = no). In deriving this variable, we adapted the “boundary intersection” method of environmental hazard exposure measurement [64] to estimate the likelihood of block exposure to an environmental health hazard in the context of the FWC. Figure 1, Map B, shows the spatial distribution of our dependent variable throughout Flint and its nine wards. Of the land parcels surveyed, 4210 (7.51 percent) had LSL connections, and only 4.36 percent of the Flint’s 2010 land area (88,177.77 km^2^) were covered by LSL-connected parcels. Yet, as indicated in Figure 1, Map B, block exposure to LSL-connected parcels was diffuse throughout Flint. Indeed, of the Flint’s 3005 census blocks defined for the year 2010, we found that 1401 (46.62 percent) intersected an LSL-connected parcel.

### 2.3. Explanatory Variables

We developed block-level racialized and gendered single-parent family structure variables from the 2010 U.S. decennial census [57] in order to examine the intercategorical environmental health vulnerabilities at the block level. We calculated the *percentage of families* in a block that had *white*, *Black*, and *Latinx* householders who were *single parents*, *single mothers*, or *single fathers*. According to the 2010 U.S. decennial census, a “family consists of a householder and one or more other people living in the same household who are related to the householder by birth, marriage, or adoption. All people in a household who are related to the householder are regarded as members of his or her family” [58] (p. 640).

In the context of Latinx households, the U.S. decennial census data have known limitations as counts of Latinx householders are technically of “any race” and not mutually exclusive from counts of white and Black householders. However, these are the only available census data that allow us to measure the gendered family structures for racialized white, Black, and Latinx householders in a parsimonious fashion. Scholarly [47] and journalistic accounts [65,66,67] indicate that Flint’s Latinx population identifies with a distinct Latinx identity outside the categories of white and Black.

Our own analysis of block-level 2010 census data for Flint supports prior accounts about the distinct racial identities of Flint’s white, Black, and Latinx householders and indicates parsimony among our block-level intercategorical explanatory variables. We found that the percentage of families with a Latinx householder had the highest correlation with the percentage of families with a householder that identified as “Some other race alone” (Kendall Tau-b correlation coefficient = 0.593, *p* < 0.001, N = 2202). The percentage of families with a Latinx householder had lower correlations with the percentage of families with a white householder (Kendall Tau-b correlation coefficient = 0.190, *p* < 0.001, N = 2202) and with a Black householder (Kendall Tau-b correlation coefficient = −0.294, *p* < 0.001, N = 2202). The limited correlation at the block level between the percentage of families with white, Black, and Latinx householders witnessed in our preliminary analyses carried forward into our regression analyses (see Section 3.2) where we found no concerning levels of multicollinearity among our explanatory and control variables.

### 2.4. Control Variables

We account for additional factors that are broadly associated with elevated aggregate patterns of environmental health vulnerability within the broader environmental inequality outcomes literature. Previous research has found an association between low household income levels and aggregate-level predictors of lead exposure during and beyond the FWC [7,8,14,61] using the five-year average estimates of household income from the American Community Survey (ACS), which has well-known granularity limitations [68,69].

Given the limitations of the ACS, we followed recent research that uses an alternative control for socioeconomic status that is available at the census block level: *the percentage of owner-occupied housing units* from the 2010 U.S. decennial census [70]. The choice to use this alternative measure is supported by research that demonstrates housing tenure is an important component of the social stratification system throughout the United States [71,72,73], and especially in Flint [47]. Further, housing tenure is an important correlate of unequal environmental health hazard exposure in the United States [27]. The negative association between aggregate levels of owner-occupied housing units and environmental health hazard exposure is typically attributed to the elevated socioeconomic and political standing that homeowners wield through their access to financial resources and mortgage credit, as well as homeowner associations and social and political networks that advocate on their behalf [72,74]. Within Flint, higher shares of homeowners present in a block may also be associated with a greater likelihood of LSL replacement and thus less likelihood of LSL exposure, as suggested in the prior research on the FWC [61].

We also account for the spatial dimensions of elevated environmental health vulnerability, which may be manifest in this case with aggregations of neighboring census blocks that are exposed to LSL-connected parcels. Accounting for such spatial dynamics simultaneously addresses a statistical concern over spatial autocorrelation—or how spatial effects, such as the similarity of proximate blocks, may violate the assumption of independence in these data—and a substantive concern over the extent to which LSL exposure is spatially concentrated in focal and neighboring blocks [17]. Following recent research that features spatially oriented logistic regression analyses similar to our own [70], we experimented with different distance bands of nearest neighbors (in seven-nearest-neighbor increments) to capture the sphere of influence on a focal block’s likelihood of exposure to LSL-connected parcels. Doing so is akin to incorporating a spatially lagged dependent variable into the set of independent variables within a spatial lag regression model for continuous dependent variables. Ultimately, we found that a Euclidean-based 28-nearest-neighbor threshold successfully eliminated spatial autocorrelation in the residuals of our logistic regression models and more fully represented the spatial dimensions of LSL exposure during the FWC.

Accordingly, the final control we include in our analysis is *the percentage of nearest 28 blocks that intersect LSL-connected parcels.* By integrating this variable into our analysis, we maintain that it is important to note that potential spatial effects indicate how geographic space affects the distribution of risk of exposure within this context, but does not mean that baseline models without the spatial control are not meaningful. Therefore, we discuss both baseline models and models that account for the spatial concentration of LSL exposure as key aspects of our intercategorical ecological approach.

### 2.5. Analytical Strategy

We use binary logistic regression models to test our guiding hypotheses regarding the likelihood of block exposure to LSL-connected parcels. We define our logistic regression models as:

(1) $$\log\;\left(\frac{\pi }{1-\;\pi }\right)=\alpha +\underset{k}{\sum }\beta_{k}X_{k}$$

where log(*π*/1 − *π*) is the natural log of the odds of block intersection with LSL-connected parcels, *α* is the constant, and *β* is the coefficient for the *k* number of *X* independent variables [17,19,20]. In our logistic regression analysis, we use an analytical sample of 2202 blocks with non-missing data. In accordance with the techniques used to operationalize our spatial control variable, we use a 28-nearest-neighbor, row-standardized spatial weights matrix to diagnose the degree of spatial dependence in our regression models.

## 3. Results

### 3.1. Descriptive Statistics

Table 1 displays the descriptive statistics for variables used in our analysis of 2202 census blocks. The table elaborates on Figure 1 by showing that among the 2202 blocks included in our analysis, 58 percent intersected LSL-connected parcels. Table 1 also illustrates the average prevalence (mean) and variation (standard deviation) in the degree of racialized and gendered single-parent families among blocks included in our analysis.

Similar to city-level patterns, we see that single-parent Black families constitute higher average shares of all families (37.96 percent) than single-parent white (18.93 percent) and single-parent Latinx (2.18 percent) families. Single-mother families comprise much of the single-parent white, Black, and Latinx families, as indicated in Table 1. Single-mother Black families are more prevalent, averaging 31.09 percent of families at the block level, followed by single-mother white (13.50 percent) and single-mother Latina (1.48 percent) families.

### 3.2. Likelihood of Block Exposure to LSL-Connected Parcels

**Figure 2:**
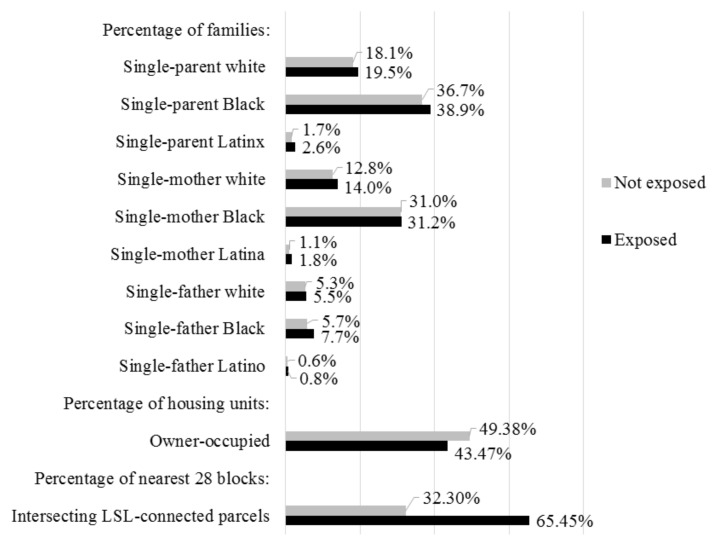
Comparison of mean block characteristics by block exposure to LSL-connected parcels.

Before summarizing our logistic regression results of the likelihood of block exposure to LSL-connected parcels, we present Figure 2 to illustrate bivariate patterns in the intercategorical complexity of block exposure to LSL-connected parcels. Figure 2 shows that the mean concentration of all racialized and gendered single-parent family structures is higher in exposed blocks versus non-exposed blocks. Noteworthy disparities manifest for three of the intercategorical variables. Blocks exposed to LSL-connected parcels had higher concentrations of single-parent Latinx families (1.53 times), single-mother Latina families (1.64 times), and single-father Black families (1.35 times) than non-exposed blocks. As for the control variables, blocks exposed to LSL-connected parcels were twice as likely to have their 28 neighbors also exposed to LSL-connected parcels, but they had lower shares of owner-occupied housing units than non-exposed blocks.

Table 2 summarizes the first set of results from our logistic regression models that address our first research aim. That is, those models estimate the likelihood of block exposure to LSL-connected parcels as a function of block-level percentages of single-parent white, Black, and Latinx families, net of the housing tenure and spatial concentration of LSL exposure controls.

In Model 1a, we found partial support for our guiding hypothesis that higher concentrations of nonwhite single-parent families would be correlated with increased likelihood of exposure to LSL-connected parcels. Similar to the results for the percentage of white single-parent families, net of other factors included in Model 1a, the percentage of single-parent Black families had no significant association with block exposure to LSL-connected parcels. However, a one-point increase in the percentage of single-parent Latinx families is significantly associated with a 2.1-percent increase in the odds of block exposure to LSL-connected parcels. As expected, the percentage of owner-occupied housing units is a negative predictor of LSL exposure in Model 1a.

The significant and positive Moran’s I diagnostic for Model 1a indicates that there is spatial clustering in the regression residuals in that model as one would expect: LSLs are clustered across Flint in ways that are unexplained in Model 1a. Including the percentage of the nearest 28 blocks that intersect LSL-connected parcels removes the spatial dependence in the regression residuals and contributes to better model fit in Model 1b with a smaller –2 log likelihood and higher pseudo R^2^ value. Accounting for additional spatial dynamics with the spatial control increases the p-value of the percentage of single-parent Latinx families variable coefficient (*p* = 0.062), but the magnitude of the effect is still comparatively large. Accordingly, Model 1b provides marginal support for the nonwhite single-parent family hypothesis and suggests that elevated odds of LSL exposure occur through spatially dependent mechanisms.

Consistent with our discussion of the parsimony of our independent variables in Section 2.3, we found that there are no alarming instances of multicollinearity among the explanatory and control variables input into Models 1a and Models 1b. In Model 1a, we found only a moderate positive correlation (r = 0.669) between the percentage of single-parent white families and percentage of single-parent Black families. In the better performing Model 1b, that moderate positive correlation decreased (r = 0.537). Further, we found in supplemental logistic regression analyses that the results presented in Table 2 did not substantively change when the models excluded the moderately correlated percentage of single-parent Black families variable (see Appendix A: Table A1) or the percentage of single-parent white families variable (see Appendix A: Table A2). Thus, we are confident that the results presented in Table 2 are not substantively affected by multicollinearity.

Our second model series (Models 2a and 2b) are shown in Table 3. Those models address our second research aim, and they reveal the importance of attending to the intersection of race, gender, and single-parent family structures in analyzing the spatial dimensions of environmental health vulnerability during the FWC.

We glean two important insights from the results presented in Table 3. First, we see that when considering the spatial concentration of Latinx and Black single-parent families, the gender of the single parent matters. Second, the effects become greater in magnitude (and statistically significant) when we consider gender because the effect for each is now considered separately from the apparent null effects of the percentage of single-mother Black and single-father Latino families on the odds of block exposure to LSL-connected parcels.

The results for single-mother Latina and single-father Black families are illustrative of these broader takeaways from our intercategorical ecological approach reflected in comparisons of Table 3 and Table 2. We see that net of the expected negative effect of the percentage of owner-occupied housing units and other racialized and gendered single-parent family measures, the effect detected in Model 1a (Table 2) regarding the percentage of single-parent Latinx families results from the separate inclusion of the percentage of single-mother Latina families in Model 2a (Table 3). Further, the percentage of single-father Black families was positively associated with the odds of block exposure to LSL-connected parcels in Model 2a. In contrast, the percentage of single-parent Black families in Model 1a is not significantly associated with block LSL exposure.

Inserting the spatial control continues to affect the logistic regression results. As in Models 1a and 1b, we see that the spatial concentration of LSL exposure addresses the problem of spatial dependence in the residuals of Model 2a and contributes to better model fit in Model 2b. However, in contrast to Model 1b, one of the intercategorical variables—percentage of single-father Black families—maintains a significant effect at the *p* < 0.05 threshold on the likelihood of block exposure to LSL-connected parcels, net of the spatial control and other variables included in Model 2b. In that model, a one-point increase in the percentage of single-father Black families has a 1.1-percent increase in the odds of block exposure to LSL-connected parcels. It is noteworthy, as well, that the percentage of single-mother Latina families has the second largest effect next to the spatial control in Model 2b and that this effect is only slightly above the *p* < 0.05 threshold at *p* = 0.054.

We found once again that our regression results presented in Table 3 are not substantively affected by multicollinearity between our independent variables. In Model 2a, only the correlation between the percentage of single-mother white families and the percentage of single-mother Black families reached a moderate but not concerning level (r = 0.537). Likewise, that positive correlation was only moderate in Model 2b (r = 0.519). We then found in supplemental logistic regression analyses that the results presented in Table 3 are robust to the exclusion of the moderately correlated percentage of single-mother Black families variable (see Appendix A: Table A3) or the percentage of single-mother white families variable (see Appendix A: Table A4).

Comparing the –2 log likelihood (and pseudo R-squared) statistics for the models presented in Table 2 and Table 3 indicates that Model 2b has the most explanatory power in predicting the odds of block exposure to LSL-connected parcels. Accordingly, of the factors included in our analysis, we conclude that block exposure to LSL-connected parcels amidst the FWC was most consistently associated with the spatial concentration of LSL-connected parcels and elevated percentages of single-father Black families. Importantly, the percentage of single-mother Latina families in a block was also associated with exposure to LSL-connected parcels at levels just outside the *p* < 0.05 level.

The results from the logistic regression analyses suggest that differences in the intercategorical exposure to risk operate through the clustering of LSLs within Flint with the exception of single-father Black families who remain uniquely exposed to risk even when controlling for spatial dependence. Put differently, we find evidence that blocks with high concentrations of single-parent Latinx families (notably, single-mother Latina families) and single-father Black families were particularly likely to experience elevated exposure to LSLs during the FWC. However, much of this inequality is explained by the spatial concentration of such racialized and gendered single-parent families in clusters or “hot spots” [17] of blocks with high concentrations of LSL-connected parcels.

The unexpected result that the concentration of single-mother Black families was not associated with increased likelihood of exposure to LSL-connected parcels may be explained by returning to the raw values presented in Figure 2. Single-mother Black families were by far the most common of the gendered and racialized single-parent family structures observed, and perhaps due to this high prevalence it is unsurprising that these families occupied exposed and unexposed census blocks throughout the city of Flint. This provides an important and subtle insight: even though many single-mother Black families were (and likely still are) exposed to LSL-connected parcels in Flint, their relatively even distribution throughout the city means that the concentration of such families was not significantly associated with heightened risk of exposure.

## 4. Discussion

The present study illuminates how gender, family structure, and spatial context conditioned the racial patterning of lead exposure during the FWC in several ways. Firstly, our results indicated that single-parent Latinx families (Model 1a), and particularly single-mother Latina families (Model 2a) were associated with elevated environmental health vulnerability in baseline models that did not adjust for spatial autocorrelation. These elevated vulnerabilities were particularly concerning given how the needs of the Latinx community in Flint were consistently overlooked as the crisis unfolded. The prioritization of information is one way that controlling images may be revealed as certain groups of people are deemed more important and others are more easily ignored. For instance, a series of journalists’ accounts across the political spectrum in 2016 exposed the plight of Flint’s Latinx residents, and of Latina mothers in particular [65,66,67]. Much of that coverage focused on Flint’s Eastside. Though the crisis began in April 2014, prior to February 2016, “all official public messaging about the crisis was in English”, and no local Spanish media existed in Flint to warn the predominantly Spanish-speaking Latinx community about the lead contamination [65].

One Latina mother in Flint’s Eastside was interviewed by a journalist after having received for the first time a Spanish-printed version of the lead advisory in February 2016, nearly two years after the FWC began. Amidst tears and dread over the toxic lead exposures experienced by her young children during the FWC, especially her one-year-old infant who had likely consumed toxins in utero, she reported: “As a mother I don’t want to believe that I hurt the baby that was inside me. Why didn’t they say anything?...If they would have told us the water was contaminated we would have done things differently” (quoted in Carstensen [65]). Thus, in addition to the FWC disproportionately impacting single-mother Latina families, the informational needs of these multiply marginalized residents were also overlooked, ensuring that many were unable to take precautions in order to reduce their exposure to the city’s drinking water.

Secondly, we found that the spatial clustering of LSLs plays a key role in the distribution of risk in the case of Flint. Differences in intercategorical exposure to risk primarily, but not exclusively, operate through the ecological conditions shaping how LSLs were built and replaced (or not) within Flint. These conditions derive from historical and contemporary circumstances and depend, in part, on the geographic scale of analysis to include broader or narrower spatial extents [17]. For example, the inclusion of exurban and suburban communities surrounding Flint as featured in prior research [8] would likely draw a starker contrast between the spatial inequalities in vulnerability of exposure for different intercategorical groups.

Thirdly, we found that the percentage of single-father Black families was a consistent intercategorical predictor of block exposure to LSL-connected parcels even when controlling for spatial autocorrelation. This finding supported our guiding hypothesis regarding the elevated environmental health vulnerability of single-father Black families in Flint. However, it is important to understand this result in the context of the null finding for single-mother Black families. Such unexpected results for single-mother Black families may be due to the ubiquity and predominance of single-mother Black families generally throughout Flint, as we note above, and across the city’s census blocks (see Table 1). Those dynamics may combine to nullify the statistical association between the percentage of single-mother Black families and exposure to LSL-connected parcels. To be clear, single-mother Black families are prevalent, and they have and continue to experience multiple modes of marginalization within Flint [47] and throughout the United States [24,33,34,35]. They also tend to experience disproportionate environmental and human health conditions nationwide [17,20,36,75,76,77]. Yet, as our analyses illustrate (see Figure 2 and Table 3), the concentration of single-mother Black families was high yet nearly identical across exposed and non-exposed blocks. Because single-mother Black families were common in census blocks across the city, this measure is a poor predictor of differences in exposure within the city.

**Figure 3:**
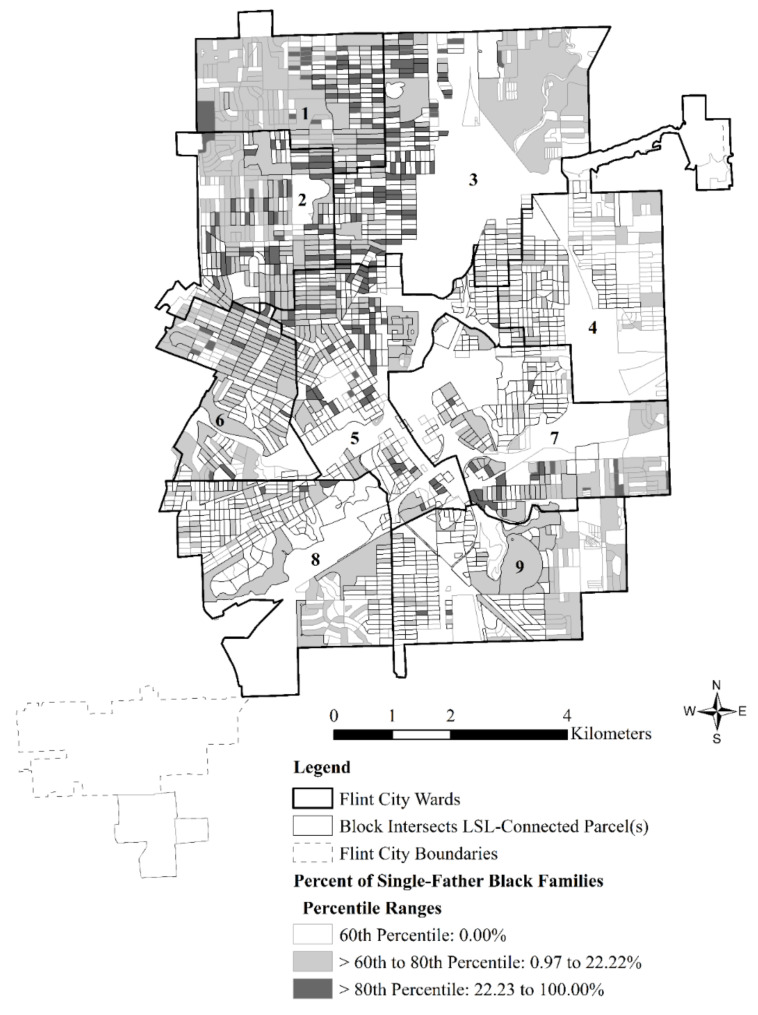
Flint wards, block intersection with LSL-connected parcels, and percentiles of the percentage of single-father Black families for 2202 blocks included in the logistic regression analysis.

In contrast, the less prevalent shares of single-father Black families were more consistently concentrated in blocks that were exposed LSL-connected parcels. Figure 3 visualizes this relationship in relation to Flint’s city wards that have been the subject of prior research. Importantly, Figure 3 shows that elevated concentrations of single-father Black families in north and west Flint overlap to some extent with wards 5, 6, and 7 that were previously identified by Hanna-Attisha et al. [8] as having elevated WLLs and BLLs during the FWC. Thus, block-level percentage of single-father Black families emerged as a particularly vulnerable aggregate-level racialized and gendered family structure during the FWC.

**Figure 4:**
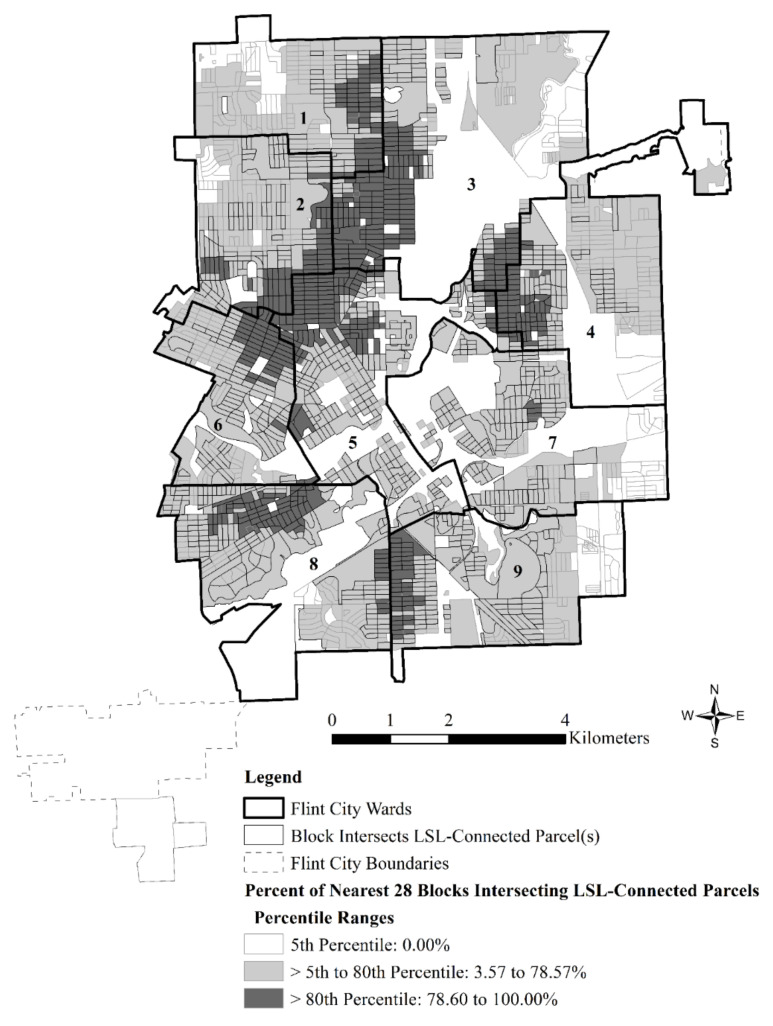
Flint wards, block intersection with LSL-connected parcels, and percentiles of the percentage of the nearest 28 blocks that intersect LSL-connected parcels for 2202 blocks included in the logistic regression analysis.

Just as important, however, is the broader spatial context in which those vulnerable blocks reside during the FWC. In fact, logistic regression analyses showed that the most consistent predictor of block exposure to LSL-connected parcels among all the factors included in the regression analyses was the extent to which neighboring blocks were also exposed to LSL-connected parcels. As shown in Figure 4, large, contiguous spans of blocks were exposed to LSL-connected parcels in north and west Flint where single-father Black families were found during the FWC (compare with Figure 3), as well as in other vulnerable sections of the city like Flint’s Eastside that is predominated by single-mother Latina families.

Our results also contribute to the growing literature on the racial ecology of lead exposures outside of Flint, particularly those finding heightened risk for Black children and families in large cities, like Detroit and Chicago [6,7]. Yet, questions remain as to how race intersects with other established environmental health vulnerabilities of gender and single-parent family structure to shape the ecology of lead exposure in diverse ways within the iconic cases of Detroit and Chicago. We argue our intercategorical ecology approach and block level of analysis offer an important avenue to address such questions in Flint and other U.S. urban spaces grappling with the legacy of lead pipes and unequal exposures in their drinking water systems [4,5,6,7].

### Limitations

Despite its merits and novel findings, several data limitations, possibly addressable in future research, are worth acknowledging. Firstly, the estimates of LSL-connected parcels as of 7 November 2016 [15] are possibly conservative. Secondly, we did not analyze block exposure to other water service lines composed of galvanized steel, copper, plastic, and/or other materials. Future research should address this set of limitations by exploring alternative data sources that may provide less conservative estimates of parcels connected to LSLs and other water service lines as of 7 November 2016 or earlier. Similar sets of analyses could also be conducted on the spatial distribution of block exposure to lead and other water service line connections throughout subsequent phases of Flint’s water service line replacement program, starting, for example, with the replacement of 6228 lead and galvanized steel lines as of 18 December 2017 [78]. Such lines of inquiry could provide a fuller understanding of the diversity of block exposure to water service lines and thus correlates of “environmental privilege” [79] and disadvantage during the FWC.

Thirdly, future research could build on this study by considering additional explanatory factors and measures of intercategorical environmental health vulnerabilities. We recommend that future research analyzes census block-level exposure differentials by the racialized and gendered statuses of single-parent families with differently aged children who had elevated exposures and adverse health outcomes during the FWC [8,13]. In addition, future research could move beyond Latinx, Black, and white family structures to assess the effects of block levels of single-parent family structures for other racialized groups, such as Asian Americans, Pacific Islanders, and Indigenous peoples. In the process, we recommend that such work keeps in mind that those additional racialized groups represented about 1 percent of the Flint’s population in 2010. Further, future research could replace the intercategorical variables we used with homeowner and renter status for white, Black, and Latinx households to advance our understanding of how the intercategorical complexity of housing tenure is associated with the likelihood of block exposure to lead and other water service lines, net of single-parent family households and the spatial concentration of exposures to various water service lines.

Fourthly, future qualitative studies could develop this intercategorical framework further. They could do so while building on Desmond [71,80] to explore how “structural constraints” (i.e., low income status, housing costs, and the presence of children) and racialized and gendered “interaction patterns” between renters and landlords concentrate multiply marginalized renters and families in environmentally hazardous housing in Flint. We anticipate that housing tenure (i.e., renter vs. homeowner status) is likely a key avenue by which controlling images are deployed in a manner that produces, reproduces, and legitimates how intersecting racialized and gendered environmental health vulnerabilities manifested during the FWC.

Fifthly, we did not attend to the individual-level health-related outcomes of lead exposure during the FWC because such a focus predominates in previous studies of the FWC [8,13,14]. Instead, we sought to address the research gap in the literature on the aggregate-level intercategorical and spatial dynamics of lead exposure in Flint, a key mechanism affecting health outcomes. However, future research could build on this study and previous individual-level studies of FWC-related health outcomes by using multilevel approaches emphasized in recent studies of the racial ecology of lead exposure and poisoning in other U.S. cities [6,7] and in Krieger’s [81,82] ecosocial theory of population health. Both of these approaches draw our attention to the multilevel and historical nature of environmental health vulnerability. Krieger [81,82], in particular, identifies acute and chronic exposure to environmental hazards as one of the key pathways of embodiment through which we literally come to embody our lived experiences by incorporating the material and social into our biology. Sampson and Winter [7] refer to this embodiment as “toxic inequality,” resulting from early and repeated toxic exposures to lead that are patterned by various axes of social and spatial division. We found significant inequalities in exposure to LSL-connected parcels among blocks with heightened concentrations of single-mother Latina families and single-father Black families in Flint. Future research should examine how individual-level health inequalities previously found in Flint [13] reflect the toxic and intercategorical inequality that structures Flint’s recent drinking water contamination crisis.

Despite these limitations, our intercategorical ecological approach and rigorous spatial analysis makes visible fine-grained and complex environmental health vulnerabilities that have been neglected in prior research and public accounts of the FWC.

## 5. Conclusions

Discrimination and lack of concern for the welfare of the Flint’s predominantly Black population are widely cited as influential factors placing Flint at heightened risk of exposure to the contaminated drinking water supply during the FWC [10,11]. Thus, the FWC has emerged as a quintessential contemporary example of environmental racism, which has had disastrous environmental health implications for many of Flint’s residents. Importantly, Flint’s residential settlements were not all exposed to lead hazards equally. Indeed, paralleling broader sociological studies outside of Flint [4,6,7], research is beginning to shed light on (1) Flint’s complex racial ecology of lead exposure and (2) the significance of single-parent families in contributing to environmental health vulnerability during the FWC [8,14].

The present study builds on the previous research on the FWC and most other research on family structure and environmental health and inequality outcomes [29,30]. Specifically, our novel intercategorical ecology model follows in the footsteps of Liévanos’ [17,20] intercategorical approach to analyzing spatial inequalities in carcinogenic air pollution cluster exposure, particularly for settlement spaces of Black and Latinx households and families. In addition, this study offers a unique “angle of vision” [24] into how gender, family structure, and spatial context condition the racial patterning of lead exposure within Flint that was yet to be identified in prior research. A long history of discrimination, deployment of controlling images, and spatial strategies such as loan underwriting procedures that favored multiply privileged residents contributed to shaping the geospatial landscape of Flint prior to the onset of the water crisis. This history was therefore inscribed in spatial patterns of vulnerability to such a crisis and accounts for why some multiply marginalized populations were more likely concentrated in Flint census blocks that had elevated exposures to LSLs.

These findings draw attention to how family structure, race, and gender intersect within the context of vulnerability to lead exposure and provide insight into controlling images about what constitutes a family at risk and assumptions about who lives where in U.S. cities. These assumptions can leave residents, like Latinx parents in this case, without the information that they need to protect their families. We hope that our findings help to focus future scholarship and corrective actions by regulators, public health officials, activists, and community members on the multiply marginalized women, men, families, and settlement spaces that experienced heightened risk of toxic exposures.

## Figures and Tables

**Table 1:** Descriptive statistics for variables used in the logistic regression analysis of block exposure to LSL-connected parcels (N = 2202).

Variable	Mean	Standard Deviation
*Dependent variable*:		
Blocks intersecting LSL-connected parcels	0.58	0.49
		
*Explanatory variables*:		
Percentage of families:		
Single-parent white	18.93	23.39
Single-parent Black	37.96	32.73
Single-parent Latinx	2.18	6.73
Single-mother white	13.50	18.41
Single-mother Black	31.09	28.82
Single-mother Latina	1.48	5.19
Single-father white	5.43	11.13
Single-father Black	6.87	12.25
Single-father Latino	0.71	3.96
		
*Control variables*:		
Percentage of owner-occupied housing units	46.78	23.42
Percentage of the nearest 28 blocks intersecting LSL-connected parcels	52.21	28.44

**Table 2:** Results from logistic regression of block exposure to LSL-connected parcels on racialized single-parent family structure, socioeconomic status, and the spatial clustering of LSL exposure (N = 2202 blocks).

Variables	Model 1a			Model 1b		
	B ^1^	SE ^2^	OR ^3^	B	SE	OR
Percentage of families:						
Single-parent white	0.001	0.003	1.001	0.002	0.003	1.002
Single-parent Black	0.001	0.002	1.001	0.002	0.002	1.002
Single-parent Latinx	0.021 **	0.008	1.021	0.015 ^†^	0.008	1.015
						
Percentage of owner-occupied housing units	−0.011 ***	0.002	0.989	−0.005	0.003	0.995
Percentage of the nearest 28 blocks intersecting LSL-connected parcels				0.058 ***	0.002	1.060
						
Constant	0.732 ***	0.185	2.079	−2.534 ***	0.262	0.079
						
Pseudo R-squared	0.031			0.469		
–2 log likelihood	2944.710			2050.179		
Model chi-square	50.753 ***			945.283 ***		
Degrees of freedom	4			5		
Moran’s I of regression residuals ^4^	0.358 ***			−0.001		

^1^ Unstandardized regression coefficients; ^2^ standard error of regression coefficients; ^3^ odds ratios; ^4^ Moran’s I test of residuals conducted with 9999 permutations and a 28-nearest-neighbor spatial weights matrix. ^†^ Correlation is significant at the 0.1 level (two-tailed). ** Correlation is significant at the 0.01 level (two-tailed). *** Correlation is significant at the 0.001 level (two-tailed).

**Table 3:** Results from logistic regression of block exposure to LSL-connected parcels on racialized and gendered single-parent family structure, socioeconomic status, and the spatial clustering of LSL exposure (N = 2202 blocks).

Variables	Model 2a			Model 2b		
	B ^1^	SE ^2^	OR ^3^	B	SE	OR
Percentage of families:						
Single-mother white	0.002	0.003	1.002	0.002	0.004	1.002
Single-mother Black	−0.002	0.002	0.998	0.000	0.003	1.000
Single-mother Latina	0.026 **	0.010	1.026	0.022 ^†^	0.011	1.022
Single-father white	−0.001	0.005	0.999	0.001	0.006	1.001
Single-father Black	0.015 ***	0.004	1.015	0.011 *	0.005	1.011
Single-father Latino	0.014	0.012	1.014	0.007	0.013	1.007
						
Percentage of owner-occupied housing units	−0.011 ***	0.002	0.989	−0.005	0.003	0.995
Percentage of the nearest 28 blocks intersecting LSL-connected parcels				0.058 ***	0.002	1.060
						
Constant	0.752 ***	0.185	2.122	−2.533 ***	0.263	0.079
						
Pseudo R-squared	0.039			0.472		
–2 log likelihood	2929.894			2044.823		
Model chi-square	65.568 ***			950.640 ***		
Degrees of freedom	7			8		
Moran’s I of regression residuals ^4^	0.354 ***			−0.001		

^1^ Unstandardized regression coefficients; ^2^ standard error of regression coefficients; ^3^ odds ratios; ^4^ Moran’s I test of residuals conducted with 9999 permutations and a 28-nearest neighbor spatial weights matrix. ^†^ Correlation is significant at the 0.1 level (two-tailed). * Correlation is significant at the 0.05 level (two-tailed). ** Correlation is significant at the 0.01 level (two-tailed). *** Correlation is significant at the 0.001 level (two-tailed).

**Figure A1:**
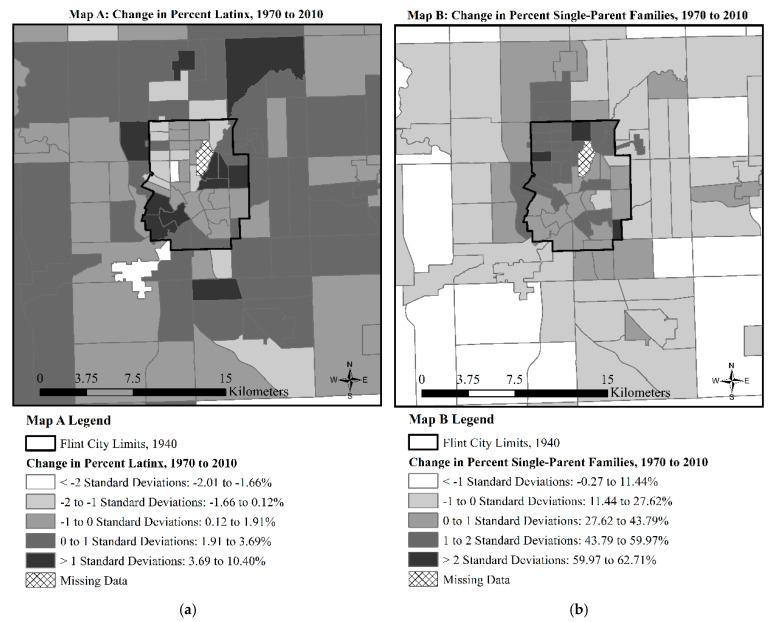
Census tract-level changes in Flint and the vicinity (i.e., Genesee County) from 1970 to 2010—a period known as “The Fall of Flint” [47]—for the percentage of (**a**) Latinx and (**b**) single-parent families. Note: authors’ original analysis of longitudinal census tract-level demographic change data [83] and historic Flint City boundaries [57].

## Data Availability

The data presented in this study, except for the University of Michigan–Flint GIS Center’s spatial data on water service line connections for Flint, Michigan, are available on request from the corresponding author. Those interested in the water service line connections data should contact the University of Michigan–Flint GIS Center.

## Appendix A

**Table A1:** Results from logistic regression of block exposure to LSL-connected parcels on the percentage of single-parent white families, percentage of single-parent Latinx families, socioeconomic status, and the spatial clustering of LSL exposure (N = 2202 blocks).

Variables	Model 1a			Model 1b		
	B ^1^	SE ^2^	OR ^3^	B	SE	OR
Percentage of families:						
Single-parent white	0.000	0.002	1.000	0.001	0.002	1.001
Single-parent Latinx	0.021 **	0.008	1.021	0.015 ^†^	0.008	1.015
						
Percentage of owner-occupied housing units	−0.012 ***	0.002	0.988	−0.005 *	0.002	0.995
Percentage of the nearest 28 blocks intersecting LSL-connected parcels				0.058 ***	0.002	1.060
						
Constant	0.834 ***	0.111	2.303	−2.408 ***	0.188	0.090
						
Pseudo R-squared	0.030			0.469		
–2 log likelihood	2945.182			2050.658		
Model chi-square	50.280 ***			944.804 ***		
Degrees of freedom	3			4		
Moran’s I of regression residuals ^4^	0.343 ***			−0.007		

^1^ Unstandardized regression coefficients; ^2^ standard error of regression coefficients; ^3^ odds ratios; ^4^ Moran’s I test of residuals conducted with 9999 permutations and a 28-nearest-neighbor spatial weights matrix. ^†^ Correlation is significant at the 0.1 level (two-tailed). * Correlation is significant at the 0.05 level (two-tailed). ** Correlation is significant at the 0.01 level (two-tailed). *** Correlation is significant at the 0.001 level (two-tailed).

**Table A2:** Results from logistic regression of block exposure to LSL-connected parcels on the percentage of single-parent Black families, percentage of single-parent Latinx families, socioeconomic status, and the spatial clustering of LSL exposure (N = 2202 blocks).

Variables	Model 2a			Model 2b		
	B ^1^	SE ^2^	OR ^3^	B	SE	OR
Percentage of families:						
Single-parent Black	0.001	0.001	1.001	0.001	0.002	1.001
Single-parent Latinx	0.021 **	0.008	1.022	0.016 ^†^	0.008	1.016
						
Percentage of owner-occupied housing units	−0.012 ***	0.002	0.989	−0.005 *	0.002	0.995
Percentage of the nearest 28 blocks intersecting LSL-connected parcels				0.058 ***	0.002	1.060
						
Constant	0.802 ***	0.130	2.229	−2.425 ***	0.207	0.088
						
Pseudo R-squared	0.030			0.469		
–2 log likelihood	2944.989			2050.642		
Model chi-square	50.473 ***			944.821 ***		
Degrees of freedom	3			4		
Moran’s I of regression residuals ^4^	0.343 ***			−0.007		

^1^ Unstandardized regression coefficients; ^2^ standard error of regression coefficients; ^3^ odds ratios; ^4^ Moran’s I test of residuals conducted with 9999 permutations and a 28-nearest-neighbor spatial weights matrix. ^†^ Correlation is significant at the 0.1 level (two-tailed). * Correlation is significant at the 0.05 level (two-tailed). ** Correlation is significant at the 0.01 level (two-tailed). *** Correlation is significant at the 0.001 level (two-tailed).

**Table A3:** Results from logistic regression of block exposure to LSL-connected parcels on the percentage of single-mother white families, percentage of single-mother Latina families, percentage of single-father white families, percentage of single-father Black families, percentage of single-father Latino families, socioeconomic status, and the spatial clustering of LSL exposure (N = 2202 blocks).

Variables	Model 3a			Model 3b		
	B ^1^	SE ^2^	OR ^3^	B	SE	OR
Percentage of families:						
Single-mother white	0.003	0.003	1.003	0.003	0.003	1.003
Single-mother Latina	0.026 **	0.010	1.026	0.022 ^†^	0.011	1.022
Single-father white	0.000	0.004	1.000	0.002	0.005	1.002
Single-father Black	0.015 ***	0.004	1.015	0.011 *	0.005	1.012
Single-father Latino	0.014	0.012	0.243	0.007	0.013	0.573
						
Percentage of owner-occupied housing units	−0.011 ***	0.002	0.989	−0.004 ^†^	0.002	0.996
Percentage of the nearest 28 blocks intersecting LSL-connected parcels				0.058 ***	0.002	1.060
						
Constant	0.639 ***	0.122	1.895	−2.564 ***	0.201	0.077
						
Pseudo R-squared	0.039			0.472		
–2 log likelihood	2930.553			2044.854		
Model chi-square	64.909 ***			950.609 ***		
Degrees of freedom	6			7		
Moran’s I of regression residuals ^4^	0.340 ***			−0.007		

^1^ Unstandardized regression coefficients; ^2^ standard error of regression coefficients; ^3^ odds ratios; ^4^ Moran’s I test of residuals conducted with 9999 permutations and a 28-nearest-neighbor spatial weights matrix. ^†^ Correlation is significant at the 0.1 level (two-tailed). * Correlation is significant at the 0.05 level (two-tailed). ** Correlation is significant at the 0.01 level (two-tailed). *** Correlation is significant at the 0.001 level (two-tailed).

**Table A4:** Results from logistic regression of block exposure to LSL-connected parcels on the percentage of single-mother Black families, percentage of single-mother Latina families, percentage of single-father white families, percentage of single-father Black families, percentage of single-father Latino families, socioeconomic status, and the spatial clustering of LSL exposure (N = 2202 blocks).

Variables	Model 2a			Model 2b		
	B ^1^	SE ^2^	OR ^3^	B	SE	OR
Percentage of families:						
Single-mother Black	−0.002	0.002	0.998	−0.001	0.002	0.999
Single-mother Latina	0.026 **	0.010	1.027	0.022 *	0.011	1.023
Single-father white	−0.001	0.005	0.999	0.001	0.006	1.001
Single-father Black	0.014 ***	0.004	1.014	0.011 *	0.005	1.011
Single-father Latino	0.014	0.012	1.014	0.007	0.013	1.007
						
Percentage of owner-occupied housing units	−0.012 ***	0.002	0.988	−0.005	0.002	0.995
Percentage of the nearest 28 blocks intersecting LSL-connected parcels				0.058 ***	0.002	1.060
						
Constant	0.830 ***	0.146	2.294	−2.439 ***	0.223	0.087
						
Pseudo R-squared	0.039			0.471		
–2 log likelihood	2930.360			2045.279		
Model chi-square	65.102 ***			950.184 ***		
Degrees of freedom	6			7		
Moran’s I of regression residuals ^4^	0.339 ***			−0.007		

^1^ Unstandardized regression coefficients; ^2^ standard error of regression coefficients; ^3^ odds ratios; ^4^ Moran’s I test of residuals conducted with 9999 permutations and a 28-nearest-neighbor spatial weights matrix. * Correlation is significant at the 0.05 level (two-tailed). ** Correlation is significant at the 0.01 level (two-tailed). *** Correlation is significant at the 0.001 level (two-tailed).
